# Quantitative Light Fluorescence (QLF) and Polarized White Light (PWL) assessments of dental fluorosis in an epidemiological setting

**DOI:** 10.1186/1471-2458-12-366

**Published:** 2012-05-20

**Authors:** Iain A Pretty, Michael McGrady, Christian Zakian, Roger P Ellwood, Andrew Taylor, Mohammed Owaise Sharif, Timothy Iafolla, E Angeles Martinez-Mier, Patcharawan Srisilapanan, Narumanas Korwanich, Michaela Goodwin, Bruce A Dye

**Affiliations:** 1 Colgate Palmolive Dental Health Unit, School of Dentistry, University of Manchester, Lloyd Street North, Manchester Science Park, England, M15 6SH, United kingdom; 2 Oral Health Unit, School of Dentistry, University of Manchester, Lloyd Street North, Manchester Science Park, England, M15 6SH, United kingdom; 3 CDC, National Center for Health Statistics, Metro IV Building, 3311 Toledo Road, Hyattsville, MD, 20782, USA; 4 NIH, National Institute of Dental and Craniofacial research, 31 Center Drive Ste 5B55, Bethesda, MD, 20892-2190, USA; 5 Indiana University, School of Dentistry, Oral Health Research Institute, 415 Lansing St, Indianapolis, IN, 46202-2876, USA; 6 Faculty of Dentistry, Chiang Mai University, T. Suthep, A. Muang, Chiang Mai, 50200, Thailand

## Abstract

**Background:**

To determine if a novel dual camera imaging system employing both polarized white light (PWL) and quantitative light induced fluorescence imaging (QLF) is appropriate for measuring enamel fluorosis in an epidemiological setting. The use of remote and objective scoring systems is of importance in fluorosis assessments due to the potential risk of examiner bias using clinical methods.

**Methods:**

Subjects were recruited from a panel previously characterized for fluorosis and caries to ensure a range of fluorosis presentation. A total of 164 children, aged 11 years (±1.3) participated following consent. Each child was examined using the novel imaging system, a traditional digital SLR camera, and clinically using the Dean’s and Thylstrup and Fejerskov (TF) Indices on the upper central and lateral incisors. Polarized white light and SLR images were scored for both Dean’s and TF indices by raters and fluorescence images were automatically scored using software.

**Results:**

Data from 164 children were available with a good distribution of fluorosis severity. The automated software analysis of QLF images demonstrated significant correlations with the clinical examinations for both Dean’s and TF index. Agreement (measured by weighted Kappa’s) between examiners scoring clinically, from polarized photographs and from SLR images ranged from 0.56 to 0.92.

**Conclusions:**

The study suggests that the use of a digital imaging system to capture images for either automated software analysis, or remote assessment by raters is suitable for epidemiological work. The use of recorded images enables study archiving, assessment by multiple examiners, remote assessment and objectivity due to the blinding of subject status.

## Background

There are a number of effective strategies available to prevent dental caries, which include the use of fluorides including community fluoridation schemes (water, salt and milk), fluoridated toothpastes, and other fluoride delivery systems, such as tablets, drops, and varnishes. The US Surgeon General has reported that community water fluoridation is an effective, safe, and ideal public health activity that benefits individuals of all ages across all socioeconomic strata [1] Indeed, the Centers for Disease Control and Prevention has listed community water fluoridation as one of the top ten public health achievements in the past century [2].

**Figure 1:**
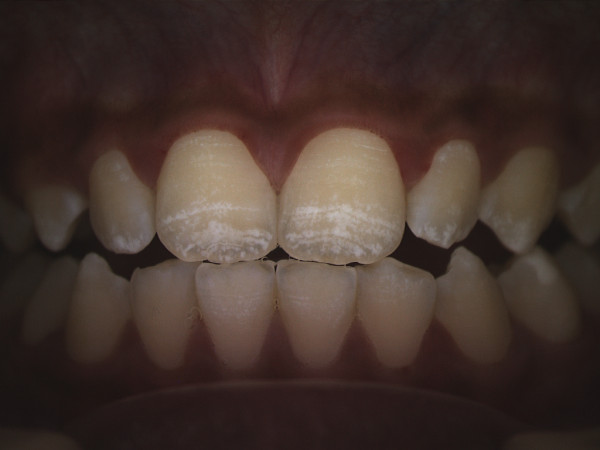
Example of mild fluorosis of the type seen in lifetime residents of optimally fluoridated drinking water communities.

**Figure 2:**
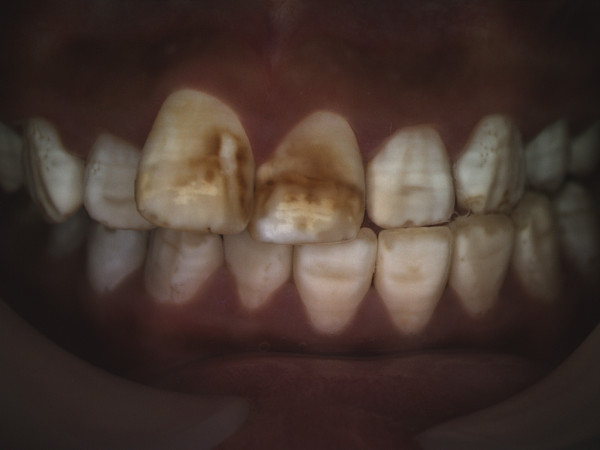
More severe dental fluorosis of the type seen in individuals living in areas with naturally high fluoride content in their drinking water.

Changes in the appearance of tooth enamel can occur if ingestion of *excessive* amounts of fluoride occurs during critical time periods during tooth development [3,4]. These changes can result in dental fluorosis, which in its mildest forms presents as areas of white striations following the developmental lines of enamel [5]. The severity of fluorosis observed is multifactorial but is strongly linked with both the amount and timing of fluoride exposure [3,4]. Figure 1 provides an example of mild fluorosis. High fluoride exposure, such as seen in areas with naturally occurring water fluoridation in excess of 1 ppm, may result in more severe presentations of fluorosis that include enamel pitting, brown discoloration and ultimately enamel loss [5] (Figure 2).

There is a need to measure the prevalence and severity of fluorosis within populations for surveillance purposes. For example in England there is a legislative obligation on those health authorities who have added fluoride to water systems to measure and report dental fluorosis prevalence. Other countries, such as the United States, have included assessments of dental fluorosis within population surveys, e.g. the National Health and Nutrition Examination Survey (NHANES) [6,7] and the National Survey of Oral Health in U.S. School Children (1986-1987) [8]. These assessments have been traditionally undertaken by clinical examiners who assign scores based upon a clinical index. Examples of indices used include Dean’s Index [9], the Fluorosis Risk Index (FR) [10], Thylstrup and Fejerskov Index (TF) [5] and the Tooth Surface Index of Fluorosis (TSIF) [11]. In the US, the Dean’s Index is predominant and has been used in NHANES for national population surveillance efforts while in Europe the TF Index is well accepted.

While these indices have been used extensively their deployment is not without criticism. Like many clinical indices, they are highly subjective and prone to bias [12-15], for example knowledge of water fluoridation status by the examiner, especially in countries where such activities are uncommon. In England, the York Centre for Reviews and Dissemination (CRD) report on the evidence supporting water fluoridation cited lack of examiner blinding as a particular weakness and potential source of significant examiner bias that could potentially lead to over estimation of dental fluorosis [13]. While it may be possible to reduce this effect by moving subjects from one location to a central examining centre, this does have obvious logistical, safety and consent issues [16].

The most common means of mitigating examiner bias is via the use of photographs. These can be taken during examinations and graded remotely, thus enabling the examiners to be blinded [17]. However, there is a lack of research looking at how such images can be standardized, their quality optimized (especially with regard to specular reflections caused by ring flashes) and their analysis recorded [18]. Collecting images as part of epidemiological studies has additional benefits including archiving, the ability to assess longitudinal changes, scoring by multiple examiners, remote examiner scoring and producing training sets for examiner calibration. A visual record of the study can also be of help for research governance reasons.

However, while photographic methods serve to address the blinding issue there are numerous other sources of potential bias in relation to the use of such indices. These include the assessment of dental fluorosis against other enamel defects, especially in populations with low dental fluorosis prevalence or severity, and the application of personal thresholds and examiner drift [17,19]. Additionally, training examiners is a complex and costly procedure and there is an acute lack of appropriately trained individuals. Therefore, there is also a need to consider if the assessment of dental fluorosis could be undertaken using an automated grading system [18].

The use of quantitative light induced fluorescence (QLF) in such a system was described by Pretty et al. in 2006 [18] when a camera based system was employed on 26 subjects to determine if both the hardware and software would enable automated quantification. Early results were encouraging. The principles of QLF are described elsewhere in detail. Briefly, there is a loss of fluorescence intensity in areas of enamel hypomineralisation, which can be measured compared to sound areas and expressed as ΔF (% fluorescence loss), the area of the effected enamel measured in mm^2^ and a composite value Δ Q reported [20-24].

The system was then deployed in a large-scale epidemiological study of some 600 children in Thailand, followed by 2000 children in the UK. Data from these studies suggested that the system was able to detect a dose–response relationship between dental fluorosis and fluoride exposure and, in the UK study, between communities with and without optimally fluoridated drinking water. In these studies a single QLF camera was employed and white light images were taken with a standard 35 mm digital SLR (Single Lens Reflex).

Such photographs are difficult to standardize in an epidemiological setting and are also prone to the effects of specular reflection. This is often a confounding factor in the assessment of such images. Polarized white light (PWL) images have no specular reflection and have been employed in dental research to examine the impact of tooth bleaching therapies [25,26]. It was therefore proposed to produce a new imaging system that combined fluorescent imaging with PWL images. This new system should be able to take the images simultaneously, or at least within seconds of each other, record them in a lossless format and be simple and rapid to employ within an epidemiological survey. The resultant white light images should be simple to score and the fluorescent images should provide sufficient discrimination between sound and fluorotic areas for an automated software system. The software should produce metrics that are strongly correlated with the clinical scores.

The aim of this current study is to report on the effectiveness of this new dual imaging system (QLF + PWL) and the reliability of the remotely graded dental fluorosis images versus the dental fluorosis scores obtained from clinical examination.

## Methods

### Study population and recruitment

The study was conducted at the University of Chiang-Mai Dental School in Thailand. The population was selected from those who had previously participated in a fluorosis study and for whom TF data were available. A purposeful sample of 190 children was undertaken to ensure that there was an appropriate distribution of TF scores in the study group. Ethical approval was obtained from the Human Experimentation Committee, Faculty of Dentistry, Chiang Mai University, Thailand (clearance number 4/2009) and from the University of Manchester Committee on Ethics on Research on Human Beings (reference number 09102). Parents and children were contacted by a letter that included: a parent information sheet, a graphical (cartoon) information sheet for children and a consent form.

Children were transported to the clinical examination site from their schools and underwent an initial screening exam to check for dental disease and to clean the teeth of gross plaque deposits with a toothbrush. Any urgent treatment need was communicated to the child’s parents or guardian. Following the screening examination, participants underwent the clinical and digital imaging assessments. First, a clinical assessment to produce a Deans index score and a TF index score for each upper incisor was completed. This was followed by the taking of an independent 35 mm white light (WL) image and then the standardized QLF and PWL images were made with the dual camera system.

### Clinical scores

Clinical scores were recorded for the right and left maxillary central and lateral incisors and entered on to standard data collection sheets. The participants were assessed by two clinical examiners who were previously trained and calibrated (RPE used the TF Index; AMM used the Deans Index).

### 35 mm photographic scores

These WL photographs were taken using a previously used technique [17]. A lip retractor was used to isolate the teeth and dried using a cotton wool roll for a period of one minute. Standardized digital images were taken with a Nikon D80 camera with a Micro Nikkor 105 mm lens and a Nikon SB 21 ring flash using only the upper illumination element. Images were captured at an angle of 15° to perpendicular in order to minimize specular reflection with a 1:1 reproduction ratio (life size). Three images were taken; a central view and left and right lateral views (to include a clear image of the canine). None of the images contained any identifying features of the participants. A photographic log form was completed to enable the digital files to be linked to the unique subject identifier.

### Dual camera images – technical set up

**Figure 3:**
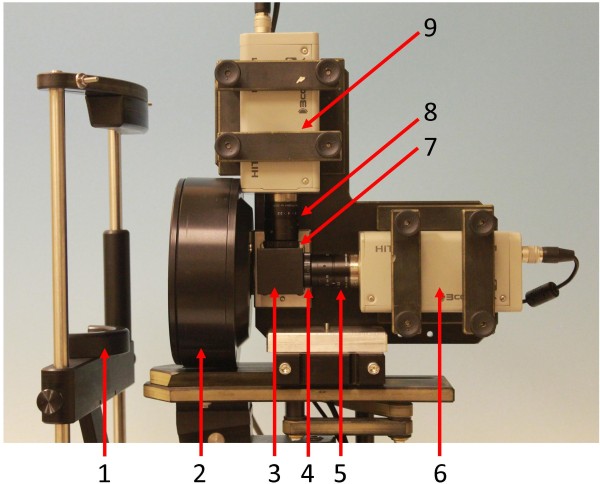
**Dual camera imaging system**. Experimental set up for QLF-WL dual-modality imaging system. 1: Patent chin rest for stabilization, 2: White and near-UV LED illumination ring, 3: beam-splitter cube, 4: linear polarizer, 5: WL imaging lens, 6: dedicated 3CCD camera for WL imaging, 7: yellow filter, 8: QLF imaging lens and 9: dedicated 3CCD camera for QLF imaging.

The dual-modality imaging was combined by means of a 50%-transmission-50%-reflection beam splitter which divides the incoming light into horizontal and vertical imaging paths with the same field of view for both PWL and the QLF imaging. Figure 3 illustrates the equipment.

Each path had a triple-charge coupled device (3CCD) colour camera (HV-F31F Hitachi Kokusai Electric Inc.) for image detection. Both imaging systems shared a common custom-made light emitting diode (LED) ring illuminator. The LED array had two internal concentric banks of 30 near-UV (ultra-violet) LEDs, each centered at 405 nm (B5-437-CVD, Roithner LaserTechnik GmbH), and two external concentric banks of 30 white LEDs, each with an emission band from 450 nm to 625 nm (B5-430-JD Roithner LaserTechnik GmbH). Undesired long-wavelength emission from the near-UV LEDs was removed by means of a blue glass filter placed in front of these LEDs. The white light illumination was polarized by means of a ring-cut linear polarizing film (45668, Edmund Optics) that was placed in front of the corresponding LEDs.

The incoming light in the WL path was cross-polarized by means of a linear polarizer (SKR FIL POL-LIN/25,5, Stemmer Imaging, Ltd.) whereas the incoming light in the QLF path was filtered by means of a 515 nm long-pass glass colour filter (45069, Edmund Optics). Images for each path were taken using a 25 mm focal length lens (TF25DA-8B, Fujinon Corporation) with a 1 mm-extension ring placed in between the lens and the camera.

To reduce motion artifacts, a custom-made geometry stabilizer with chin and forehead rests was used in order to stabilize the participant while imaging the tooth. The cameras were connected to a laptop (Dell Latitude) via firewire for image capture and processing, and the illuminators were connected to the computer via a USB control box. The cameras and illuminators were controlled by custom-written software, which had been pre-configured with the study design. The software’s main function was to capture a white light image, switch the illuminator to near-UV, capture a QLF image with the other camera, switch the illuminator back to white-light mode, and store both of these images together, under a filename and directory path which encoded the subject ID, imaging modality and image slot. The camera technician reviewed all images after capture, and again at the end of each participant’s visit. Rejected images could then be recaptured.

Lastly, the software guided the user through the capture of grey- and colour-card images, which were used to calibrate the cameras and convert the RGB (red, green, blue) values in the images into device-independent units. These were captured in the same way as participant images. The software also used a reference card of constant reflectance and fluorescence to correct for changes in LED efficiency, say due to changing temperatures. This was done before each set of participant or calibration images were taken.

### Dual camera images – participant images

Lip retractors were placed and the teeth gently dried with compressed air. Participants then placed their chin on the imaging device’s rest and the images were taken. A total of three images sets were taken – central, left and right. The process of capturing each image takes approximately 2 s, with the subject present at the imaging station for a total of 2 – 3 min.

**Figure 4:**
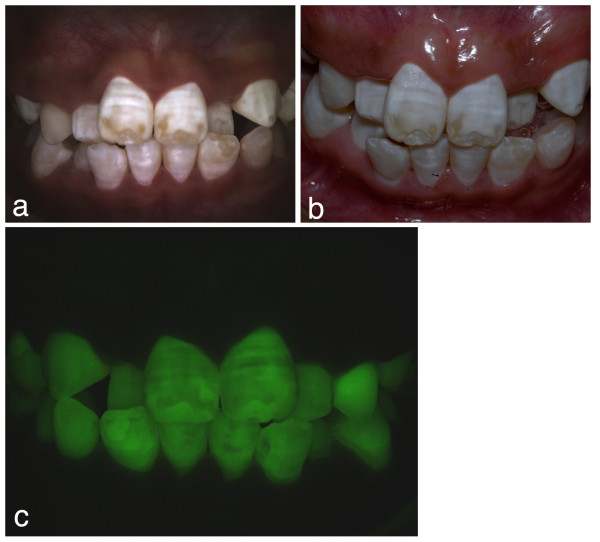
**Example of an image set collected during the study**. **a)** Polarized white light image (PWL). **b)** Traditional 35 mm image. **c)** QLF image.

**Figure 5:**
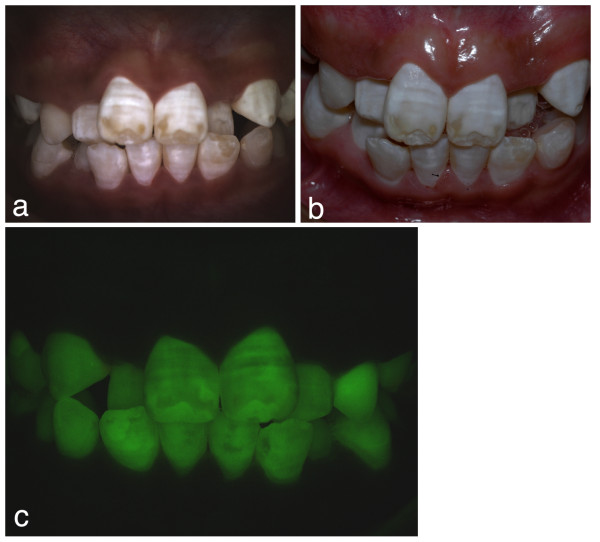
**Further example of an image set collected during the study.a)** Polarized white light image (PWL). **b)** Traditional 35 mm image. **c)** QLF image.

Example image sets are provided in Figures 4 and 5 demonstrating subjects with differing levels of fluorosis. Note the obvious specular reflection on the non-polarized white light images, and, in comparison, the lack of any reflection on either the PWL or the QLF image. The images demonstrate the field of view and arrangement of dentition captured.

### Digital image analysis

The digital white light images were exported and integrated into two presentations; one for the independent WL and one for the PWL, which was acquired from the dual imaging system. A total of 39 images in each set were duplicated for re-test analysis. These presentations were sent, with a recording sheet, to the graders (RPE & MMG for the TF Index; AMM & BAD for the Dean’s Index). No restrictions were placed on the raters in terms of their approach to the scoring. They were simply asked to score the images as they would have done clinically however timings were not standardised nor were monitor resolutions, size or contrast. The QLF images were assessed using automated software. A software “mask” was manually drawn around each central incisor to highlight the region of interest and then the automated algorithm was applied. The image analysis was undertaken using a the convex hull approach [24,27] and previously described in detail by Taylor [28]. The map of fluorescence loss (relating to areas of enamel hypomineralisation) could then be thresholded to remove background noise, with all pixels below the threshold set to zero and all those above the threshold included in the map. In this study in order to include milder forms of fluorosis the threshold was set at a level of 5 (out of 255) pixels.

### Statistical analysis and scoring

Data were entered from the paper-based records of the clinical examinations and the photographic assessments into SPSS. QLF metric data were imported directly. Various statistical techniques were used to assess the distribution of the scores and the levels of agreement between examiners, across different techniques. The distribution of dental fluorosis within the population was assessed using a combination of the Wilcoxon Test and the McNemars test. Wilcoxon was used to determine if there was any statistically significant difference between the techniques (i.e., clinical exam, PWL or WL). If a statistical difference was detected the scores were then dichotomized and McNemars was used to determine whether there were significant differences or changes between each set of scores using the related, dichotomized variables. The most common severe tooth score was derived using the mode. A Bonferroni correction was used to minimize the potential of a type 1 error when multiple comparisons were made.

Weighted Kappa’s (linear) were then used to assess the level of agreement at both examiner levels, between techniques and again between examiners for each technique. Automated evaluation of fluorosis was compared against both the clinical tooth score and PWL tooth score against QLF percentage area, ΔF and ΔQ using Spearman’s rank and Kendal’s Tau b. Finally a weighted kappa was used on repeat scores to determine the examiners reliability (test-retest) of the 39 repeated images in the photographic presentation assessment.

## Results

Data were available for 164 consenting children, 36 children were unavailable for examination as they were absent from school during the study. Additional file 1 Table S 1 presents the distribution of fluorosis within the study population (based on most severe tooth scored) but this should not be confused with population prevalence as these subjects were screened and selected to present a broad range of fluorosis scores. The table also provides information on the differences in scores between the different visual systems with significant differences being found between clinical scores and imaging modalities. This was only the case for the TF scores. To summarize Additional file 1 Table S 1, there was significant difference in scoring dental fluorosis between the clinical exam and the PWL image as well as the clinical exam and the standard WL image for examiner 1 using the TF Index. For examiner 3, using Dean’s index, there was no difference in scoring dental fluorosis between either the clinical exam and PWL or standard white light. For examiners 2 and 4, there was significant difference (Wilcoxon test) in the overall scoring of dental fluorosis between PWL and standard WL images regardless of the index used, but there was no difference at the individual fluorosis scoring level when the Bonferroni correction was applied.

Additional file 2 Table S 2 presents the agreement between the measures of fluorosis with the same examiner employing different systems (intra-examiner). Overall, the Kappa scores ranged from 0.50 to 0.57 for Examiner 1 and 0.63 to 0.65 for Examiner 3 when comparing dental fluorosis assessments made by clinical exam and digital imaging. Intra-rater reliability was consistently higher for all 4 examiners when comparing PWL to standard WL images ranging from 0.69 to 0.92. Table 1 presents the comparisons between the same tests but employed by different examiners (inter examiner). Overall, inter-rater reliability scores were higher for the TF index (ranging from 0.69-0.74) compared to the Deans index scores (ranging from 0.50 to 0.61).

**Table 1:** Agreement between assessments using Polarized White Light (PWL) and standard White Light (WL) images for dental fluorosis evaluated with linear weighted Kappa’s

	**Examiner 1 & Examiner 2**		**Examiner 3 & Examiner 4**	
	**A**	**B**	**A**	**B**
**PWL**	0.7298	0.7381	0.6100	0.5991
**WL (35 mm)**	0.6887	0.7355	0.5489	0.4970

A = Most common severe tooth scored (mode) B = Most severe tooth scored

Different examiners using same test.

Table 2 presents the results of the repeated 39 images incorporated within the presentations for PWL images based on 3 different presentations: scoring obtained from only the left central incisor, the left lateral incisor, and the highest scored tooth regardless of tooth type. These Kappa scores ranged from 0.70 to 0.89. These data demonstrate substantial to almost perfect agreement measured using linearly weighted Kappa’s [29].

**Table 2:** Test-Retest analysis of 39 Polarized White Light (PWL) images for intra-examiner agreement

	**Examiner 1***RPE*	**Examiner 2***MGM*	**Examiner 3***AMM*	**Examiner 4***BD*
**Upper left central**	0.8942	0.8570	0.8941	0.7904
**Upper left lateral**	0.6996	0.8782	0.7652	0.8496
**Highest tooth scored**	0.8075	0.8726	0.8679	0.7818

Table 3 presents the QLF metric data compared to the clinical TF and Dean’s scores (using the upper left central incisor). The mean percent area of affected dental enamel with a TF score of 0 was 17% and increased in magnitude to 43% for a TF score of 7. For the Deans index, the mean percent area of affected dental enamel increased from 15% for normal enamel appearance to 46% for severe dental fluorosis. Linear correlation (Spearman and Kendall) between the dental fluorosis levels and the change in affected dental enamel area as determined by QLF assessments was statistically significant.

**Table 3:** Automated evaluation results of the Quantitative Light Fluorescent (QLF) images for fluorosis compared to clinical exam indices tooth scores for the upper left central incisor

**QLF METRIC (mean)**	**TF SCORE (Clinical from Examiner 1)**								**Spearman’s rho**	**Kendall’s tau b**
	**0**	**1**	**2**	**3**	**4**	**5**	**6**	**7**	**P < 0.001**	
**% Area**	16.89	21.17	27.90	32.88	45.42	45.30	49.70	42.68	.735	.593
Δ **F**	0.0482	0.0540	0.0622	0.0747	0.1067	0.1049	0.0891	0.1205	.730	.586
Δ **Q**	0.0094	0.0122	0.0183	0.0258	0.0526	0.0553	0.0460	0.0514	.742	.600
**DEANS SCORE (Clinical from Examiner 3)**										
	**Normal**	**Questionable**		**Very Mild**	**Mild**	**Moderate**		**Severe**		
**% Area**	14.90	22.36		22.05	32.07	31.03		45.75	.793	.652
Δ **F**	0.0444	0.0566		0.0548	0.0669	0.0670		0.1076	.777	.635
Δ **Q**	0.0069	0.0139		0.0126	0.0224	0.0210		0.0537	.798	.659

Table 4 presents the same comparisons but using the assessment of polarized white light images as the gold standard, again for the upper left lateral incisor and similar results were obtained. The mean percent area of affected dental enamel with a TF score of 0 was 17% and increased in magnitude to 43% for a TF score of 7. For the Deans index, the mean percent area of affected dental enamel increased from 14% for normal enamel appearance to 48% for severe dental fluorosis. Linear correlation between the dental fluorosis levels and the change in affected dental enamel area was also statistically significant, but does appear to be higher for the QLF-PWL comparison (Table 4) as oppose to the QLF-clinical exam results (Table 3).

**Table 4:** Automated evaluation results of the Quantitative Light Fluorescent (QLF) images for fluorosis compared to scores obtained from the Polarized White Light (PWL) images for the upper left central incisor

**QLF METRIC (mean)**	**TF SCORE (PWL from Examiner 1)**								**Spearman’s rho**	**Kendall’s tau b**
	**0**	**1**	**2**	**3**	**4**	**5**	**6**	**7**	**P < 0.001**	
**% Area**	17.20	16.50	22.89	30.10	44.12	49.67	42.31	42.71	.782	.644
Δ **F**	0.0530	0.0470	0.0560	0.0660	0.0950	0.1216	0.0843	0.0922	.738	.600
Δ **Q**	0.0125	0.0082	0.0135	0.0203	0.0468	0.0654	0.0371	0.0394	.780	.641
**DEANS SCORE (PWL from Examiner 4)**										
	**Normal**	**Questionable**		**Very Mild**	**Mild**	**Moderate**		**Severe**		
**% Area**	13.78	13.79		18.44	25.98	34.24		48.03	.873	.726
Δ **F**	0.0446	0.0418		0.0517	0.0606	0.0732		0.1106	.808	.656
Δ **Q**	0.0062	0.0061		0.0097	0.0164	0.0270		0.0590	.861	.713

## Discussion

This study presents a population with a range of fluorosis severity that is typically not seen in Western populations [30]. This distribution facilitates the testing of a new assessment system for surveillance of dental fluorosis across populations with differing levels of fluoride exposure. As presented in Additional file 1 Table S 1, 8-12% of this study population had a Dean’s index score of 4 (severe dental fluorosis). In the United States, less than 1% of the general population is affected by severe dental fluorosis [31]. The data in Additional file 1 Table S 1 also demonstrates a recognized phenomenon; that clinical scores tend to be lower than those from photographs, especially when examining the less severe forms of dental fluorosis [17,32-34]. This is explained by the magnification, contrast and time that photographs afford to the examiner vs. the clinical examination. In this study differences were found when TF = 0 and TF = 1. Despite this the majority of the TF scores, and all of the Dean’s score showed no significant differences in the assessment of severity using either of the manual digital imaging techniques (PWL or WL). This lends support to the notion that they are at least as effective at detecting and quantifying fluorosis as the clinical examination.

This idea is further supported by the kappa values seen in Additional file 2 Table S 2. Clinical examinations were only undertaken by Examiners 1 and 3, but the kappa values follow a similar pattern when comparing the agreement between the clinical and the images, vs. comparisons between the two imaging systems. The TF examinations achieve moderate agreement comparing clinical and photographic systems whereas the Deans comparators present substantial agreement based on the scale of agreement suggested by Landis and Koch [34][29].

In general the two manually scored photographic components demonstrated good and consistent agreement with the clinical scores, with more discrepancies occurring towards the less severe calls. The impact of examiner time, size of image and appearance under either polarized or flashlight have been offered as explanations for this difference. It is also likely the personal thresholding will impact on these data – which is more apparent at less severe calls [17]. The use of photographs may reduce this as more time is permitted to assess the image. The test – retest scores were high suggesting a reliable assessment of images by the examiners.

The automated assessment of fluorosis is an ambitious aim. From a software analysis perspective a tooth has a complex curved surface that complicates the assessment of fluorescence loss and makes simple thresholding impossible [35]. The process is further complicated by the fact that any mineral loss in the enamel will present as a decrease in fluorosis; therefore caries, some enamel defects, restorative materials and even extrinsic stain are all potential confounders for the system. Indeed, the difference between detection and diagnosis is clear in this application – the system is only detecting fluorescence loss and this is a proxy measure for fluorosis.

It is therefore surprising to see the highly significant correlations between the TF and Dean’s score for each of the QLF metrics using either a clinical or photographic gold standard. The correlations proved to be unaffected by the severity of the fluorosis and a clear relationship between clinically determined disease and ΔF, ΔQ and area can be seen. These data suggest that the automated system, using a hand-drawn mask to delineate the region of interest, followed by a convex hull algorithm, has substantial utility in the objective assessment of enamel fluorosis. The process of mask drawing is a simple one, taking approximately 30 s for each tooth. A trained non-clinician can undertake this task.

While encouraging, it should be recognized that the QLF data have been derived from the central incisors only. These teeth are readily imaged and present less convexity than the canine teeth and are less prone to displacement from the depth of field than the laterals. However, data from earlier studies suggest that assessment of the central incisor alone is sufficient to separate populations of fluoridated and non-fluoridated communities (reference?). Another potential limitation of using digital images as proposed by our system is that dental fluorosis assessments will be limited to those teeth in the “aesthetic zone,” that is, the upper anteriors (canine to canine), and may therefore underestimate the true epidemiological prevalence of fluorosis in a population. Nevertheless, the impact of fluorosis is, largely, aesthetic in nature and would resonate with the general population given the increasing attention directed by many western populations towards aesthetic dentistry. Traditionally, surveillance efforts have focused on anterior and posterior teeth. NHANES for example has assessed for dental fluorosis by examining all permanent teeth except for third molars. Furthermore, surveillance systems based on a limited set of teeth could be useful--even if biased-- if the same criteria is applied consistently.

Although our findings indicate that using digital images to assess for dental fluorosis has validity when compared with clinical measures, these results are preliminary. Additional research is required to understand what would be the impact of a surveillance system that limits assessing dental fluorosis to just upper anterior teeth only, what would be the impact of incorporating QLF readings from canines and lateral incisors into an overall assessment of dental fluorosis, and how using PWL and QLF images for surveillance may be further confounded by the age of subjects [35].

## Conclusion

This study lends further support to the use of photographs for remote scoring of dental fluorosis. The ability to control for examiner bias, enable multiple examiners and provide archived evidence of the examinations are all important benefits of the process. Health technicians (or dental auxiliaries) could function as the camera technician, providing an example of skill mix usage and possibly facilitating epidemiological studies and surveillance of fluorosis by reducing the cost associated with in-field clinical examiners.

PWL images reduce the incidence of specular reflection and while they were not superior to their traditional 35 mm counterparts they can be taken *without* a 15° incline (required on the traditional camera to avoid flash reflection). This facilitates the use of an automated system and decreases the need for photographic training.

The automated QLF system demonstrated significant correlations with clinical and photographic gold standards, and while additional information is required on the use of multiple teeth within the algorithm the results support the use of the system within epidemiological programmes as a useful data adjunct.

The dual camera system was simple to operate and was well tolerated and accepted by all subjects. The ability for the device to quickly capture near simultaneous images of high and consistent quality suggests that the imaging methodology is appropriate for epidemiologic studies assessing for dental fluorosis.

## Abbreviations

ΔF: Delta F – the % change in fluorescence; ΔQ: Delta Q – the composite metric of DF and area in mm2; CCD: Charged coupled device (camera); CRD: Centres for Review and Dissemination; LED: Light emitting diode; NHANES: National Health and Nutrition Examination Survey; PWL: Polarized white light image; QLF: Quantitative light induced fluorescence; RGB: Red, green and blue; SLR: Single lens reflex; TF: Thylstrup and Fejerskov Index; TSIF: Tooth Surface Index of Fluorosis; UV: Ultra-violet; WL: A standard white light image obtained from a 35 mm digital SLR camera.

## Competing interests

None of the authors are aware of any competing interests in the production of this manuscript. The University of Manchester Dental Health Unit is co-funded by Colgate Palmolive.

## Authors’ contributions

IAP prepared the protocol, undertook study monitoring, and wrote the manuscript. MMG was an examiner and provided input into the data analysis. CZ designed the optical components of the system. RPE was a clinical and photographic examiner and contributed to the design of the camera system. AT designed the study software. MOS drew the masks for the QLF analysis. BAD was a photographic examiner and contributed to the protocol development and manuscript. TI was a study monitor and assisted with the data analysis. AMM was a clinical and photographic examiner. PS & NK were local investigators and undertook clinical and photographic examinations. MG analyzed the data, produced the report tables and contributed to the writing of the manuscript. All authors read and approved the final manuscript

## Disclaimer statement

The findings and conclusions in this report are those of the author(s) and do not necessarily represent the official position of the Centers for Disease Control and Prevention.

## Pre-publication history

The pre-publication history for this paper can be accessed here:

http://www.biomedcentral.com/1471-2458/12/366/prepub

## Supplementary Material

Additional file 1: Table S1 Distribution of dental fluorosis within the study population (most severe tooth scored) Click here for file

Additional file 2: Table S2 Agreement between assessments using Polarized White Light (PWL) or standard White Light (WL) images with the clinical exam for measures of dental fluorosis evaluated with linear weighted Kappa’s Click here for file
