# Supplementary material for: Quantitative Light Fluorescence (QLF) and Polarized White Light (PWL) assessments of dental fluorosis in an epidemiological setting
Source: BMC Public Health. 2012 May 20;12:366. doi: 10.1186/1471-2458-12-366 (PMC3490889; doi:10.1186/1471-2458-12-366)
Supplement: Additional file 1: Table S1 — Distribution of dental fluorosis within the study population (most severe tooth scored) [file 1471-2458-12-366-S1.doc]

**TABLE 1 – Distribution of dental fluorosis within the study population (most severe tooth scored)**

| **TF INDEX** | **0** | **1** | **2** | | **3** | **4** | | **5** | **6** | | **7** | Wilcoxon Test | | |
| --- | --- | --- | --- | --- | --- | --- | --- | --- | --- | --- | --- | --- | --- | --- |
| Examiner 1 Clinical | 33% | 20% | 24% | | 11% | 4% | | 5% | 2% | | 1% | Z = -6.972,  p<0.001 |  | Z = -6.386 |
| Examiner 1 PWL | 9% | 31% | 27% | | 18% | 3% | | 6% | 6% | | 1% |  | Z = -1.280, p>0.05 |  |
| Examiner 1 WL (35mm) | 10% | 33% | 26% | | 15% | 7% | | 5% | 3% | | 3% |  |  | P<0.001 |
| Examiner 2 PWL | 16% | 28% | 29% | | 18% | 2% | | 4% | 2% | | 1% | Z = -4.207, p<0.001 |  |  |
| Examiner 2 WL (35mm) | 16% | 21% | 27% | | 19% | 3% | | 6% | 4% | | 3% |  |  |  |
|  | | | | | | | | | | | | | | |
| **DEAN’s INDEX** | **Normal** | **Questionable** | | **Very Mild** | | | **Mild** | **Moderate** | | **Severe** | | Wilcoxon Test | | |
| Examiner 3 Clinical | 25% | 7% | | 37% | | | 17% | 3% | | 12% | | Z = -0.146,  p>0.05 |  | Z = -0.160, |
| Examiner 3 PWL | 16% | 8% | | 45% | | | 20% | 6% | | 6% | |  | Z = -1.268,  p>0.05 |  |
| Examiner 3 WL (35mm) | 16% | 5% | | 48% | | | 18% | 7% | | 6% | |  |  | p>0.05 |
| Examiner 4 PWL | 9% | 12% | | 26% | | | 20% | 23% | | 10% | | Z = -3.278, p<0.001 |  |  |
| Examiner 4 WL (35mm) | 4% | 13% | | 21% | | | 26% | 28% | | 8% | |  |  |  |

Examiner 1 RPE, Examiner 2 MGM
Examiner 3 AMM, Examiner 4 BD

|  | *Indicates a statistically significant difference between Clinical and PWL using McNemar’s test (*Bonferroni correction) |
| --- | --- |
|  | *Indicates a statistically significant difference between Clinical and 35mm WL using McNemar’s test (*Bonferroni correction) |
| *Please note even though Wilcoxon indicated a significant difference for examiner 2 and examiner 4 (PWL vs. 35mm WL) there was no significant difference seen at each scoring level due to the Bonferroni correction* | |
