# Supplementary material for: Quantitative Light Fluorescence (QLF) and Polarized White Light (PWL) assessments of dental fluorosis in an epidemiological setting
Source: BMC Public Health. 2012 May 20;12:366. doi: 10.1186/1471-2458-12-366 (PMC3490889; doi:10.1186/1471-2458-12-366)
Supplement: Additional file 2: Table S2 — Agreement between assessments using Polarized White Light (PWL) or standard White Light (WL) images with the clinical exam for measures of dental fluorosis evaluated with linear weighted Kappa’s [file 1471-2458-12-366-S2.doc]

**TABLE 2 – Agreement between assessments using Polarized White Light (PWL) or standard White Light (WL) images with the clinical exam for measures of dental fluorosis evaluated with linear weighted Kappa’s**

|  | **TF Index Examiner 1** | | **TF Index Examiner 2** | | **Deans Index Examiner 3** | | **Deans Index Examiner 4** | |
| --- | --- | --- | --- | --- | --- | --- | --- | --- |
|  | **A** | **B** | **A** | **B** | **A** | **B** | **A** | **B** |
| **Clinical exam vs. PWL** | 0.5507 | 0.5373 | XX | XX | 0.6586 | 0.6371 | XX | XX |
| **Clinical exam vs. WL (35mm)** | 0.5073 | 0.5758 | XX | XX | 0.6518 | 0.6522 | XX | XX |
| **PWL vs. WL (35mm)** | 0.6871 | 0.7259 | 0.7017 | 0.7115 | 0.9238 | 0.9185 | 0.7162 | 0.7464 |

A = Most common severe tooth scored (mode) B = Most severe tooth scored

*Same examiner using different assessments*
